# The Role of Preference on Outcomes of People Receiving Evidence-Informed Community Wound Care in Their Home or in a Nurse-Clinic Setting: A Cohort Study (*n* = 230)

**DOI:** 10.3390/healthcare2030401

**Published:** 2014-09-19

**Authors:** Margaret B. Harrison, Elizabeth G. VanDenKerkhof, Wilma M. Hopman, Meg E. Carley

**Affiliations:** 1 School of Nursing, Queen’s University, 92 Barrie Street, Kingston, ON K7L 3N6, Canada; E-Mails: ev5@Queensu.ca (E.G.V.); meg.carley@queensu.ca (M.E.C.); 2 Department of Anesthesiology, Queen’s University, Kingston, ON K7L 2V7, Canada; 3 Clinical Research Centre, Kingston General Hospital, Kingston, ON K7L 2V7, Canada; E-Mails: hopmanw@KGH.KARI.NET; 4 Department of Public Health Sciences, Queen’s University, Kingston, ON K7L 3N6, Canada

**Keywords:** patient preference, community wound-care, leg ulcers, community clinics, homecare

## Abstract

This study followed a cohort of community-dwelling individuals receiving wound-care in a large urban-rural region. During a randomized control trial (RCT) evaluating outcomes of receiving care in a nurse-clinic or at home, many approached were willing to participate if they could choose their location of care. This provided a unique opportunity to enroll them as a “choice” cohort, following them in the same manner as the trial participants but allowing them to select their setting of care. The objective was to investigate the role of preference and location of care on care outcomes, including satisfaction with care, healing, health-related quality of life (HRQL), pain, and resource use. This is a secondary analysis of a prospective cohort of 126 individuals enrolled in an RCT to receive care at home or in a nurse-clinic (Allocated group), and an additional 104 who received care at home or in a nurse-clinic based on their preference (Choice group). Mobile individuals with a leg ulcer of venous or mixed venous etiology, referred for community leg ulcer care, were eligible. Specially-trained nurses provided care to both groups using an evidence-informed protocol. Baseline data included socio-demographic, circumstance-of-living and a detailed wound assessment. Mean age of the cohort was 68 years. Satisfaction, healing, recurrence, pain, HRQL, and resource utilization did not differ between groups. If available, individuals should have an option of care venue given almost half of those approached indicated a clear preference for clinic or home. With outcomes being similar, health care planners and decision-makers, as well as individuals and their families, can feel confident that the setting of care will not impact the outcomes. However, larger studies in other contexts are needed to explore the interaction between choice and setting.

## 1. Introduction

Community care has become an increasingly important element within health care systems everywhere. As an example in Canada, acute care facilities have downsized, with more conditions managed and procedures performed on an outpatient basis. One advantage to this is that it may allow individuals to receive care closer to home if they prefer. An exemplar population receiving the majority of their care in the community are those suffering from chronic wounds. In the case of leg ulcers, much effort has been dedicated to evaluating “best practices” including high compression bandages [1,2,3,4] and where and how care is delivered, e.g., in the home, nurse clinics or specialist clinic [5,6,7,8,9,10,11,12]. On the other hand, individuals’ preference of where they receive their health care, or if having one’s choice makes any difference to outcomes, has typically not been a focus of research.

In a unique opportunity, we were able to follow a cohort of community dwelling individuals receiving wound care in a large urban-rural region in Ontario. It began as a randomized controlled trial (RCT) evaluating the outcomes of nurse-clinic *vs.* home delivery of evidence-informed care delivered by well-prepared providers [13]. After initiating the trial, many of those approached were willing to join the study *but* only if they could choose their preference of care location. We elected to enrol them in a “Choice Cohort” and follow them in exactly the same manner as the trial participants to assess if having choice made a difference to outcomes [14]. By combining the two previous studies for the current analysis, we have been able to not only describe a larger cohort of individuals receiving care, but also explore the role of preference on various outcomes. From a program planning perspective, we sought to understand if those with a stated preference differed from those allocated to where care is delivered. We posited that having one’s preference might improve outcomes such as satisfaction with care, well-being and quality of life and possibly the time-to-healing. From a health services perspective, there was interest in offering wound care in nurse clinics as well as home visiting, thus this analysis would also allow us to revisit the question of outcomes from care in a clinic location contrasted with home delivery in a larger sample (*n* = 230) than in our previous study (*n* = 126) [13] and in a sample that may be more representative of the target population than would be expected in a randomized controlled trial.

## 2. Experimental Section

### 2.1. Design

This study is a combined secondary analysis of a prospective cohort of 126 individuals enrolled in an RCT to receive care at home or in a nurse-clinic (Allocated Group), and an additional 104 who also received care at home or in a local community nurse-clinic, but received their care based on their preference (Choice Group). All 230 participants were followed until one-year post healing in an identical manner, using the same time points and outcome measurements. For the purpose of this analysis, we compared the Allocated and Choice groups to assess whether having choice impacted various outcomes, regardless of whether care was delivered in the home or clinic. The original study was reviewed for ethical compliance and approval obtained from the Ottawa Health Research Institute Ethics Board (#20000272-01H). A summary of key aspects of the study methodology are provided as full details are discussed elsewhere [13,14].

### 2.2. Setting and Sample

The study population for both the Allocated and the Choice Group came from the same large urban-rural Ontario region overseen by two regional homecare authorities. Inclusion and exclusion criteria for the two groups were the same: mobile individuals with a leg ulcer below the knee of venous or mixed venous and arterial etiology, with no major contraindication for clinic care (e.g., not being able to leave an ill spouse), referred for community leg ulcer care, were eligible to participate. Etiology was determined based on a thorough clinical assessment and ankle brachial pressure index (ABPI) via handheld Doppler. No upper limit for ABPI was set as criteria for exclusion. Individuals who were cognitively impaired, too ill, or unable to travel outside the home were excluded.

### 2.3. Procedures

Specially trained registered nurses performed a comprehensive, standardized clinical assessment on all individuals referred to the regional community care service for leg ulcers. Eligible individuals were provided information regarding the clinic *vs.* home trial and invited to participate. Consenting individuals were randomized to be given care in either their home or a nurse clinic and this comprised the Allocated Group. For those who expressed a willingness to participate but *not be randomized* to where care was received, they provided a modified consent for the Cohort Study noting they would have their choice of location of care. Those who chose their care setting comprised the Choice Group.

The same nursing team delivered care in both the home and clinic settings and practice was guided by international evidence-informed recommendations [6,15]. The Practice Guideline Evaluation and Adaptation Cycle (PGEAC) [15,16,17] guided development of the study’s leg ulcer care management protocol. It was prepared by an interdisciplinary task force and feedback on the draft protocol was sought from homecare nurses and family physicians [18,19] prior to implementation. The protocol was kept up-to-date through ongoing scheduled reviews [20]. Community care nurses involved in the study received additional training in leg ulcer assessment and compression bandaging application, and were familiar with the evidence for practice supporting the guideline recommendations. Compression bandaging was applied by the same nursing team in both home and clinic settings. Visits for leg ulcer care were typically scheduled two to three times per week and bandages changed based on nurses’ clinical judgment and individual circumstances (e.g., amount of exudate). Once healed, participants were advised to wear compression stockings.

### 2.4. Data Collection and Management

At the time of initial assessment following referral for community care, baseline data were gathered through interview, clinical assessment and chart review. This included socio-demographic, circumstance-of-living and a detailed wound assessment. Ulcer size was measured every 3 months until complete healing, or until 12 months post study entry, whichever came first. If healing occurred between these measurement intervals, this was recorded and the next full assessment carried out according to the schedule. Integrity of the trial and cohort study was ensured through rigorous and systematic quality assurance procedures [21,22,23].

### 2.5. Outcome Measurement

Satisfaction with care was assessed with a 12-item questionnaire developed in consultation with frontline clinicians and administered at 3-months post-baseline. Formal validation was not undertaken, as these items were based on simple statements relating to care, and the use of expert consensus generally provides a high degree of face and content validity. The questionnaire provided data on an individual’s perception of the continuity of care, information about prevention and self-managing the leg ulcer themselves, and their satisfaction with the care they received in either the clinic or home setting.

The principal healing outcome for the cohort was healing at 3-months (≤91 days). Change in ulcer size and sustainability of healing (days to first recurrence) were also monitored. Both the pain and health related quality of life (HRQL) measures were selected based on our previous work [24,25] and that of Walters *et al.* [26]. Pain was assessed using the McGill Short Form Pain Questionnaire (SF-MPQ) [27,28,29]. HRQL was assessed with the Medical Outcomes Trust SF-12^®^ [30], which measures eight self-reported aspects of HRQL, including physical function, role physical, bodily pain, general health, vitality, social function, role emotional and mental health. The SF-12 generates a Physical Component Summary (PCS) and Mental Component Summary (MCS) that are standardized to a mean of 50, with a score above and below 50 representing better and poorer than average function, respectively. Important for our health services partners, Canadian population-based normative data are also available for this measure [31]. The EuroQol (EQ-5D™) [32,33] measured aspects of functional autonomy (*i.e.*, self-care, usual activities, mobility). The EQ-5D index was derived using Canadian based population weights [34].

### 2.6. Analyses

The primary data analysis sought to describe outcomes of satisfaction with care, healing, HRQL, pain, and resource use by group (Allocated *vs.* Choice). Analysis was based on intention-to-treat; all participants were included in the analysis regardless of compliance with the location of care allocated or chosen or whether participants adhered to the care plan with compression therapy. Participants with no assessment post-baseline were excluded from analyses pertaining to healing and recurrence outcomes and those who never healed were excluded from analyses pertaining to recurrence. Kaplan-Meier survival curves were constructed for the two groups, and the statistical significance of the differences was tested using the log rank test. The proportion of individuals in each group who healed within 3 months (91 days) and recurrence rates were compared using Chi squared tests. Mean differences in self-reported health status outcomes (SF-12, pain) were compared using the independent samples t-test of either the pooled or separate variance estimates as appropriate. Variables with a non-normal distribution were analyzed with the appropriate non-parametric procedures, Mann-Whitney for unpaired data and Wilcoxon for paired data. The potential for non-response bias was assessed by comparing characteristics of those who completed and those who did not complete the SF-MPQ, SF-12, EQ-5D, and Satisfaction questionnaires.

## 3. Results

**Figure 1:**
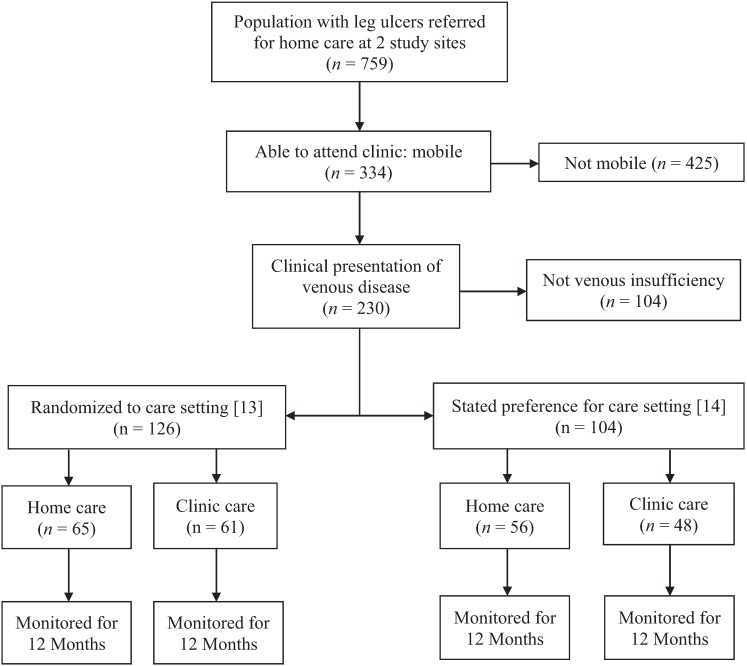
Allocated and choice, clinic and home, leg ulcer cohort study recruitment.

**Figure 2:**
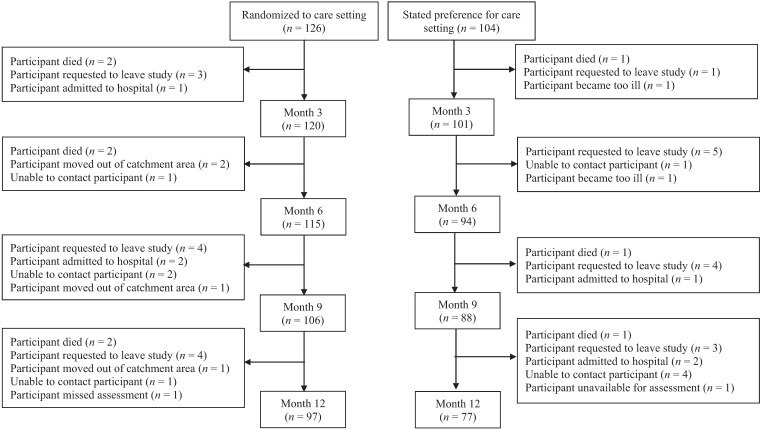
Participant flow over 12 month follow-up period.

Seven hundred and fifty-nine individuals referred for community leg ulcer care underwent a multi-step screening process (Figure 1) over a span of 28 months for the Clinic *vs.* Home RCT [13] and the Choice Cohort Study [14]. Individuals were first approached for the trial but if they stated a preference for either clinic or home, they were considered ineligible for the trial and invited to be enrolled in the Choice Cohort. Of those screened, 44% were assessed as eligible to receive clinic care due to sufficient mobility and ability to travel outside their homes. A clinical assessment followed with 69% (*n* = 230) presenting with venous disease or mixed (venous and arterial etiology) and being eligible for management with compression bandages. When approached, 55% (*n* = 126) agreed to be randomly allocated to a home or clinic care setting. However, 45% (*n* = 104) indicated a willingness to be studied but declared a preference for receiving care in one setting or the other. The combined group (Allocated for the RCT and Choice) formed the Preference Cohort of 230 individuals in the current analysis. The flow of participants over the 12 month follow-up period is illustrated by Figure 2.

There were no significant differences between the allocated and the choice group on socio-demographic, circumstance of living, health-related quality of life or clinical characteristics at baseline, *i.e.*, admission to care (Table 1). Mean age of the full cohort was 68 years. There were slightly more women (51%) than men and the majority was English-speaking (84%). Half (50.4%) had at least one previous episode of ulceration and 57.4% on admission had a current ulcer ≤5 cm^2^ for 6 months or less. Baseline SF-12 PCS scores were poor; much lower than the Canadian norm (35.7 *vs.* 51.7). The SF-12 MCS was comparable to Canadian normative values (49.4 *vs.* 50.5) [31]. In tracking adherence to the evidence-informed protocol, there were no differences found in key aspects of care received by the groups (Table 1).

### 3.1. Individual’s Satisfaction with Care

The vast majority were very or quite satisfied (95%) with the care received in the past 12 weeks and the information they received on how to care for their leg ulcers (97%), with 94% indicating that they would recommend it to others (Table 2). No differences in waiting time were observed when comparing the allocated and choice groups. Anecdotally, we recorded information from nurses about what individuals said about their preference for care. For some people, getting out to a clinic setting provided social contact which was otherwise not available to them. Others liked being booked for a specific appointment time because with home visiting they did not know when a nurse was going to come which was problematic for those still working. For some, receiving care at home was preferential because of difficulty making the arrangements to travel to the clinic, such as transportation, parking and distance to walk, and leaving a spouse or family member alone at home.

**Table 1:** Comparison of the baseline characteristics of the study population and those allocated or given a choice of care setting.

Characteristics ^1^	TOTAL (*n* = 230)	ALLOCATED Care Setting (*n* = 126)	CHOICE of Care Setting (*n* = 104)	*p*-Value
Setting of Care				
○ Home	121 (52.6)	65 (51.6)	56 (53.8)	0.79
○ Clinic	109 (47.4)	61 (48.4)	48 (46.2)	
Etiology of leg Ulcer				
○ Venous	154 (67.0)	86 (68.3)	68 (65.4)	0.67
○ Mixed	76 (33.0)	40 (31.7)	36 (34.6)	
Gender-Female	118 (51.3)	71 (56.3)	47 (45.2)	0.11
Language-English	193 (83.9)	106 (84.1)	87 (83.7)	0.99
Living Alone	87 (37.8)	54 (42.9)	33 (31.7)	0.10
Independently Mobile	167 (72.9)	89 (71.2)	78 (75.0)	0.55
Ulcer Duration				
○ ≤3 Months	129 (56.1)	73 (57.9)	56 (53.8)	0.30
○ >3 to ≤12 Months	68 (29.6)	39 (31.0)	29 (27.9)	
○ > 12 Months	33 (14.3)	14 (11.1)	19 (18.3)	
Ulcer Size				
○ ≤2.5 cm²	124 (53.9)	69 (54.8)	55 (52.9)	0.66
○ 2.5 to ≤10 cm²	61 (26.5)	35 (27.8)	26 (25.0)	
○ >10 cm²	45 (19.6)	22 (17.5)	23 (22.1)	
Previous Ulceration (yes)	116 (50.4)	62 (49.2)	54 (51.9)	0.69
Ulcer Size (cm^2^) ^†^	2.4 [0.98/6.7]	2.3 [1.1/5.8]	2.4 [0.82/9.0]	1.00 ^2^
Diathesis in years ^†^	7 [3/12]	8 [3/16]	5 [2.5/9.0]	0.05 ^2^
Duration at initial assessment in weeks ^†^	11.4 [4.6/24]	10.6 [4.7/22.7]	12.1 [4.4/30.6]	0.73 ^2^
ABPI ^†^	1.07 [0.99/1.16]	1.06 [0.98/1.14]	1.08 [1.00/1.20]	0.06 ^2^
Age (years) *	68.0 (14.2)	68.5 (14.1)	67.5 (14.5)	0.62
SF12 Scores *				
○ Mental Component	49.4 (11.1)	49.7 (11.0)	49.0 (11.3)	0.64
○ Physical Component	35.7 (10.1)	35.1 (9.9)	36.4 (10.4)	0.36
Clinical Care ^3^				
ABPI completed	223 (97.0)	122 (96.8)	101 (97.1)	1.00
Compression Therapy				
○ All	208 (91.2)	118 (94.4)	90 (87.4)	0.10
○ Venous disease	143 (94.1)	82 (96.5)	61 (91.0)	0.18
○ Mixed disease	65 (85.5)	36 (90.0)	29 (80.6)	0.33

^1^ Values are frequency (percent) unless indicated otherwise; frequency values may not always total 100% due to missing data. * values are mean (s.d.); ^†^ values are median [percentiles]; ABPI = Ankle Brachial Pressure Index. ^2^ Mann-Whitney U. ^3^ 100% of clients received a comprehensive clinical assessment.

**Table 2:** Comparison at 3 months of the individual’s perception of personal issues related to leg ulcer and satisfaction with care for those allocated or given a choice of care setting.

Characteristic ^1^	ALLOCATED Group (*n* = 102)	CHOICE Group (*n* = 80)	*p*-value
	n (%)	n (%)	
ISSUES (*n* = 182)			
Some problems walking about	54 (52.9)	39 (48.8)	0.65
Some problems with washing, dressing self	16 (15.7)	14 (17.5)	0.84
Some problems performing my usual activities	50 (49.0)	44 (55.7)	0.48
Not anxious or depressed	74 (72.5)	56 (70.0)	0.27
EuroQol EQ-5D Index †	0.77 [0.70/0.84]	0.77 [0.71/0.84]	0.77 ^2^
	**ALLOCATED Group (*n* = 97)**	**CHOICE Group** **(*n* = 80)**	
CARE AND SERVICE SURVEY ( *n* = 177)			
Wait Time			
▪ Less than 30 min	86 (88.7)	72 (91.1)	0.43
▪ Waiting 30 min–1 h	9 (9.3)	7 (8.9)	
▪ Waiting 1–2 h	2 (2.1)	0 (0.0)	
Knows the name of the nurse who takes care of leg ulcer most of the time	72 (74.2)	69 (86.2)	0.06
Very/quite satisfied with information nurse provided for how to care for leg ulcer	94 (96.9)	78 (97.5)	0.65
Very/quite satisfied with information nurse provided for leg ulcer prevention	83 (85.6)	69 (87.3)	0.74
Very/quite Satisfied with nurses’ skill	91 (94.8)	78 (97.5)	0.41
Comfortable with bandages and dressings used for treatment	55 (62.5)	53 (69.7)	0.41
Very/quite Satisfied with treatment last 12 weeks	88 (93.6)	74 (96.1)	0.67
Recommend/highly recommend care you receive to others	86 (92.5)	75 (94.9)	0.55
Overall rating of the nursing care (1 = Poor to 10 = Excellent) ^†^	10 [9/10]	10 [10/10]	0.26 ^2^

^1^ Values are frequency (percent) unless indicated otherwise; frequency values may not always total 100% due to missing data. * values are mean (s.d.); ^†^ values are median [percentiles]. ^2^ Mann-Whitney U.

### 3.2. Healing

**Figure 3:**
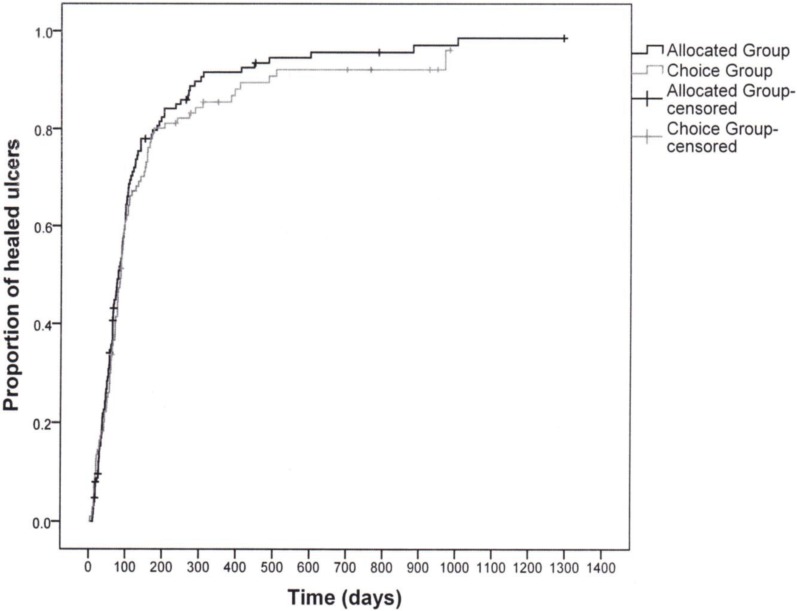
Kaplan-Meier curves showing proportion of ulcers healed by group.

Healing rates did not differ between groups, with 57.5% of the allocated group and 56.9% of the choice group being healed at 3 months (Table 3). The unadjusted Kaplan-Meier curves revealed no significant differences in the distribution of cumulative healing times between groups (log rank χ^2^ = 0.851, *p* = 0.34) (Figure 3). Similar results were found for time-to-healing. The mean time was 118 days (median 73) in the allocated group and 117 days (median 77) in the choice group. The durability of healing was derived through the recurrence rates within one year; these rates were 25.2% in the allocated group compared to 19.4% (*p* = 0.4) in the choice group (see Table 3).

### 3.3. Pain and Health Related Quality of Life

At 3 months, the number of reports of “no pain” were similar between the allocated and choice groups (Table 3). For health related quality of life, neither the MCS nor the PCS were statistically significantly different between the groups.

Resource Use: The median number of visits per week was the same for the two groups, at two visits. The remaining resource variables including total number of nursing visits overall, weeks on service, or expenditures on personnel and supplies were slightly higher for the Choice group, but did not come close to statistical significance (Table 3).

Missing Data: Survey data were missing for 17% of participants (*n* = 39). With a few exceptions, there were no statistically significant differences in baseline characteristics between those with and without missing data. Those with at least one completed survey were more likely to be female (55%, *p* = 0.02), English-speaking (86%, *p* = 0.03), with longer ulcer duration at baseline (median 12 weeks *vs.* 6 weeks, *p* = 0.01), and slightly older (mean age 69 *vs.* 63, *p* = 0.04) than those who did not complete any surveys.

**Table 3:** Healing, pain, and quality of life outcomes and resource utilization for those allocated or given a choice of care setting.

Outcome ^1^	ALLOCATED Care Setting (*n* = 126)	CHOICE of Care Setting (*n* = 104)	*p*-Value
	n (%)	n (%)	
Healing ^2^			
○ 3-month (≤91 days)	69 (57.5)	58 (56.9)	1.00
○ Recurrence rate in one year ^3^	29 (25.2)	18 (19.4)	0.40
Pain at 3 Months			
○ No pain	58 (57.4)	49 (59.8)	0.94
○ Mild/Discomfort	32 (31.7)	24 (29.3)	
○ Distressing/horrible/excruciating	11 (10.9)	9 (11.0)	
SF12 Scores at 3 Months *			
○ Mental Component	52.8 (10.4)	52.5 (11.1)	0.85
○ Physical Component	39.0 (11.3)	40.1 (12.4)	0.55
General Health Assessment			
○ Excellent or Very Good	29 (29.3)	26 (32.5)	0.32
○ Good	39 (39.4)	37 (46.2)	
○ Fair or Poor	31 (31.3)	17 (21.2)	
Resource Utilization for an Episode of Leg Ulcer Care ^4^			
○ Number of Nursing Visits ^†^	23 [12/48]	25 [14/51]	0.40
○ Weeks on Service ^†^	13 [7/24]	14 [8/27]	0.38
○ Visits per Week ^†^	2 [1.6/2.3]	2 [1.7/2.5]	0.34
○ Nursing Costs ^†^	$1135 [612/2347]	$1283 [698/2556]	0.33
○ Cost of Wound Supplies ^†^	$531 [251/1115]	$545 [191/1163]	0.97

^1^ Values are frequency (percent) unless indicated otherwise; frequency values may not always total 100% due to missing data. * values are mean (s.d.); ^†^ values are median [percentiles]. ^2^ Six clients in the Allocated group and two clients in the Choice group were not included in the analysis because of loss to follow-up after baseline. ^3^ Clients who were lost to follow-up after baseline or never healed were not included in the analysis (Allocated group n = 115, Choice group n = 93). ^4^ Time on service until leg ulcer was healed. *p*-values for resource utilization are based on Mann-Whitney U.

## 4. Discussion

This is the first study to explore differences in characteristics and outcomes of individuals receiving community leg ulcer care who were allocated to care setting *vs.* those who had their choice of care location. The community care was provided through either a home visiting or a nurse-clinic. Participants were followed in the same manner and received the same evidence-informed care by a team of specially trained nurses in both settings. Satisfaction, healing, recurrence, pain, HRQL and resource outcomes did not differ between the groups.

Interestingly, on admission to community care nearly half (45%) had a stated preference of where they would like to receive their care. Of those expressing preference, 54% wanted care in their homes while 46% elected clinic care. Given the proportion of people having a preference and the relatively balanced numbers preferring clinic or home, it may be worth healthcare authorities’ consideration when assigning people to community care if both clinic and home delivery are available. Although RCTs are thought to not represent target populations generally, this study seems to refute that argument.

Satisfaction with care, healing, recurrence, pain, and HRQL outcomes were similar between the allocated and choice groups. At baseline, the groups showed some borderline differences in diatheses and ABPI scores. This might impact the results although in our experience an ABPI of 1.06 compared to 1.08 would not be considered clinically important for decision-making. In comparison to what has been reported in the literature, healing rates in this cohort were relatively high. This likely is attributed to consistency in the delivery of evidence-informed care by trained nurses.

When comparing results from our randomized trial on clinic *vs.* home delivery [13] to the choice cohort study [14], those who chose the clinic setting for their care fared somewhat better than those who were allocated (67% compared to 58% healed by 3 months), whereas those who were allocated to receive their care at home fared slightly better than those who stated a preference for homecare (57% compared to 48% healed by 3 months), though these differences were not significant. One might contemplate based on the above, that people who choose clinic are more mobile/healthy while those who choose home are less mobile/more co-morbidities. We did not see a significant difference in these characteristics at baseline. However, the allocated group had slightly larger ulcers with longer duration and even though not statistically significant, may have been clinically relevant with their choice of setting. Clearly larger studies are needed to explore the interaction between choice and setting if this is an important issue for individuals, providers and health systems.

These results have several implications for individuals with leg ulcers, their families, health service planners and decision-makers. Given outcomes are similar with respect to satisfaction with care, healing, recurrence, pain, HRQL, resource use, whether people were allocated or had their stated their preference for care in a home or clinic setting, it seems reasonable that planners and decision-makers can feel confident in offering a choice of their care setting. The caveat here of course is that expectation of similar outcomes will take place in the context of delivery using an evidence-informed approach by trained health care providers. Similarly, if given the choice, individuals and their families can feel confident that the venue they choose for their care will not have a negative impact on their outcomes.

Leg ulcer management in the home and clinic settings each come with their own set of advantages and disadvantages. Supplies are stocked and readily available in the clinic, whereas there may be a delay in receiving supplies ordered to the home. In clinic, only supplies required for an episode of treatment can be utilized whereas in the home, returning unused supplies may not be viable due to infection control issues. Individuals who receive their care at the clinic can be given a specific appointment time. However, treatment at home might be optimal for those with mobility issues or needing to care for a spouse or family member at home. In the clinic, nurses may have an advantage of better coordination with family physicians or specialists because of access to electronic resources (*i.e.*, computer, email, fax machine), whilst in the home setting, nurses may have a better sense of a client’s lifestyle requirements and can tailor their treatment accordingly. For health service providers, client accessibility, protocols for infection control, and overhead costs are issues that need to be considered in terms of clinic care while travel time and fuel costs are important factors to consider with home delivery.

With increasing pressure on homecare resources and nursing hours considered a scarce resource, access to clinic care for individuals who are mobile or with good transportation support, could be an important consideration by authorities and health service providers. In the Canadian context, factors such as the size and distribution of the population regionally, varying urban-rural mix, and vastly varying climate from one region to another may make offering choice of clinic or home delivery not feasible. However, knowing that the quality of care delivered in either setting can result in similar outcomes should be reassuring.

## 5. Limitations of the Study

This study—a combined analysis of data from an RCT and Cohort study of those who were allocated and chose their care setting, respectively—has some limitations to take into consideration. First, inclusion criteria for the overall study was the ability to be able to attend a clinic for care; therefore, the study participants may not be representative of the population receiving community leg ulcer care given their mobility. Both the RCT and Cohort Study were conducted in two south-eastern Ontario health regions and may not be representative of a broader geographic region. However, they did represent an urban rural mix and typical characteristics of a leg ulcer population.

Nurses providing care were involved in the collection of data. Blinding the nurse to the setting was not possible. Conversely, outcomes were assessed in a rigorous and consistent manner by a small team of devoted specially trained nurses regardless of setting. Finally, this study was conducted as a combined analysis of common indicators of community leg ulcer care (satisfaction, healing, HRQL, resource use) and was not powered *a priori* to determine factors associated with choice and receiving care in either of the settings, nor was it a randomized controlled design focused on preference, thus readers are cautioned when drawing conclusions from this study. However, the results do provide a useful overview of this patient population and will be useful to those planning a larger future study. An important element for a future study would be accurate accounting of travel time incurred by nurses delivering homecare and the overhead expenditures of the nurse clinics.

## 6. Conclusions

This study examined outcomes of groups who were allocated to a setting of care (community clinic or home setting), or who had the choice of setting. In spite of the above limitations, it is our belief that if available, individuals should have an option of care venue where possible given that almost half of those approached (45%) indicated a clear preference for clinic or home. Besides our previous work where the Choice cohort [14] was examined separately from the Allocated group (RCT) [13], there have been no published studies examining the interaction between choice and setting of community care for people with leg ulcers. For planners and decision makers, taking into account the population profile, local contextual factors, resource availability, and patient perspective will aid in making the appropriate health-services decision.

Our hope is that these analyses can provide a foundation for further research in this area.
